# Non-Synonymous Single-Nucleotide Polymorphisms and Physical Activity Interactions on Adiposity Parameters in Malaysian Adolescents

**DOI:** 10.3389/fendo.2018.00209

**Published:** 2018-04-27

**Authors:** Nur Lisa Zaharan, Nor Hanisah Muhamad, Muhammad Yazid Jalaludin, Tin Tin Su, Zahurin Mohamed, M. N. A. Mohamed, Hazreen A. Majid

**Affiliations:** ^1^Department of Pharmacology, Faculty of Medicine, University of Malaya, Kuala Lumpur, Malaysia; ^2^Department of Paediatrics, Faculty of Medicine, University of Malaya, Kuala Lumpur, Malaysia; ^3^Centre for Population Health (CePH), Faculty of Medicine, University of Malaya, Kuala Lumpur, Malaysia; ^4^Sports Medicine Unit, Faculty of Medicine, University of Malaya, Kuala Lumpur, Malaysia; ^5^Department of Social and Preventive Medicine, Faculty of Medicine, University of Malaya, Kuala Lumpur, Malaysia; ^6^Department of Nutrition, Harvard T.H. Chan School of Public Health, Boston, MA, United States

**Keywords:** adolescent, Malaysian Health and Adolescents Longitudinal Research Team study, obesity, non-synonymous single-nucleotide polymorphism, physical activity, body fat

## Abstract

**Background:**

Several non-synonymous single-nucleotide polymorphisms (nsSNPs) have been shown to be associated with obesity. Little is known about their associations and interactions with physical activity (PA) in relation to adiposity parameters among adolescents in Malaysia.

**Methods:**

We examined whether (a) PA and (b) selected nsSNPs are associated with adiposity parameters and whether PA interacts with these nsSNPs on these outcomes in adolescents from the Malaysian Health and Adolescents Longitudinal Research Team study (*n* = 1,151). Body mass indices, waist–hip ratio, and percentage body fat (% BF) were obtained. PA was assessed using Physical Activity Questionnaire for Older Children (PAQ-C). Five nsSNPs were included: beta-3 adrenergic receptor (ADRB3) rs4994, FABP2 rs1799883, GHRL rs696217, MC3R rs3827103, and vitamin D receptor rs2228570, individually and as combined genetic risk score (GRS). Associations and interactions between nsSNPs and PAQ-C scores were examined using generalized linear model.

**Results:**

PAQ-C scores were associated with % BF (β = −0.44 [95% confidence interval −0.72, −0.16], *p* = 0.002). The CC genotype of ADRB3 rs4994 (β = −0.16 [−0.28, −0.05], corrected *p* = 0.01) and AA genotype of MC3R rs3827103 (β = −0.06 [−0.12, −0.00], *p* = 0.02) were significantly associated with % BF compared to TT and GG genotypes, respectively. Significant interactions with PA were found between ADRB3 rs4994 (β = −0.05 [−0.10, −0.01], *p* = 0.02) and combined GRS (β = −0.03 [−0.04, −0.01], *p* = 0.01) for % BF.

**Conclusion:**

Higher PA score was associated with reduced % BF in Malaysian adolescents. Of the nsSNPs, ADRB3 rs4994 and MC3R rs3827103 were associated with % BF. Significant interactions with PA were found for ADRB3 rs4994 and combined GRS on % BF but not on measurements of weight or circumferences. Targeting body fat represent prospects for molecular studies and lifestyle intervention in this population.

## Introduction

Childhood obesity is a cause of much concern in Malaysia, a multiethnic country in South-East Asia, as the prevalence was estimated to be the highest in the region (22% in boys and 19% in girls) (1). An increased in health problems is foreseeable as the prevalence of overweight and obesity in Malaysian adults were already among the highest in the Asian region (1). Lifestyle factors such as eating behavior (2), sugary drink consumptions (3), lack of physical activity (PA) (4), and poor sleep quality (5) have been previously shown to be associated with increased risk of obesity in the Malaysian adolescents. Only a few studies have examined the genetic contribution to obesity in this population, one gene that had been studied being the neuropeptide-Y gene (6). Thus, further studies to examine possible associations of different single-nucleotide polymorphisms (SNPs) and obesity is timely.

Non-synonymous single-nucleotide polymorphisms (nsSNPs) have been widely studied in different diseases, while some were found to be associated with obesity in different populations. nsSNPs are SNPs with single amino acid substitution in a protein sequence that may lead to missense mutation or nonsense mutation. The change in protein sequent may subsequently affect protein structure and its protein interactions and exert possible functional effects (7). Among the nsSNP associated with adiposity is the fatty acid-binding protein-2 (FABP2) rs1799883 whereby the amino acid substitution from alanine to threonine was associated with increased sensitivity to carbohydrates (8). Another is the ghrelin (GHRL) rs696217 in which amino acid substitution from leucine to methionine may increase susceptibility to obesity and metabolic syndrome. The melanocortin-3-receptor (MC3R) missense mutation Val81Ile, rs3827103, was associated with increased susceptibility to obesity and may play a role in response to weight loss intervention (9–11). Other examples of nsSNPs reported to be associated with obesity included leptin (LEPR) rs1805094 (Lys656Asn) (12), beta-2 adrenergic receptor (ADRB2) rs1042714 (Glu27Gln) (13), beta-3 adrenergic receptor (ADRB3) rs4994 (Trp64Arg) (14), and vitamin D receptor (VDR) rs2228570 (Met1Thr) (15). Rarer nsSNPs such as the peroxisome proliferator-activated receptor gamma (PPARG) rs1800571 with gain-of-function mutation (Pro115Gln) has also been found to be possibly pathogenic for morbid obesity (16). These nsSNPs are thus relevant candidates to be studied in relation to obesity in our Malaysian adolescents.

Elsewhere, the interaction between genetic factors and the environment on obesity traits are increasingly being studied. Yet, studies on how genetic factors interact with environment factors are lacking in our unique population. A recent large meta-analysis examining genome-wide and PA interaction in obesity found that the effect of obesity risk loci of fat mass and obesity-associated (FTO) gene was attenuated by 30% in physically active individuals (17). There were increasing efforts to examine other possible candidate genes that may have similar effect as the FTO genes (18, 19). Less is known about the interaction of the above mentioned nsSNPs with PA in children. Studying the effect of gene and PA interaction using other adiposity parameters such as percentage body fat (% BF) from this region of the world may also potentially uncover different susceptible SNPs compared to when using body mass indices (BMI) in adolescents such as found in a study on children from New Zealand (20).

This study was aimed to examine possible associations between selected nsSNPs and adiposity parameters in the cohort of the Malaysian Health and Adolescents Longitudinal Research Team study (MyHeARTs) (21). MyHeARTs was the first longitudinal adolescent cohort study in Malaysia and among the few in South East Asia. This cohort provides a unique opportunity to study health and lifestyle factors in this region as well as genetic contribution to certain outcomes such as obesity. nsSNPs chosen were those previously shown to be associated with obesity and of possible functional significance. Different adiposity parameters such as BMI, waist–hip ratio (WHR), and % BF will be used. This study will also examine if there are interaction between these SNPs and self-reported PA in relation to adiposity for further planning of interventions of this growing obesity problem in adolescents.

## Methodology

### Study Population

This was a cross-sectional study from the second wave of the MyHeARTs in which 15-year-old participants were recruited from selected secondary schools between May 2014 and July 2014 (*n* = 1,179). Details of the MyHeARTs have been reported using a published protocol (21). The protocols were approved by the ethics committees of the University of Malaya Medical Center (MEC Ref. No: 896.34). In short, MyHeARTs was a longitudinal cohort study that recruited secondary school students from three different states in the Central and Northern Regions of Peninsular Malaysia, namely, Selangor, Federal Territory of Kuala Lumpur, and Perak. The first wave was recruited between March and May 2012 from the age of 13 years old (*n* = 1,361) (21).

### Demographics and Puberty Status

Two self-administered questionnaires were used, a Parental Questionnaire and Student Questionnaire. These questionnaires were used to obtain demographic and lifestyle information. Puberty stages were self-reported based on Tanner staging system (22).

### Assessment of PA

Self-reported PA was assessed using the validated Physical Activity Questionnaire for Older Children (PAQ-C) based on the Malay version, which has good internal consistency and acceptable validity (23, 24). The 10 items in the PAQ-C captured the levels of PA in the last 7 days including type and frequency of different selected activities, participation at separate times during the week, daily physical activities, and any unusual activities. Calculation of the PAQ-C score has been described elsewhere whereby the score ranges between 1 (low) and 5 (high) (25). Participants were classified into active (score ≥2.33) and inactive (score <2.33) (4).

### Body Composition and Weight-Related Parameters

Measurements of these parameters were performed by trained personnel including body weight (kilograms), height, and waist and hip circumferences as per MyHeARTs study protocol (21). Height was measured without socks and shoes using a calibrated vertical Seca PorTable 217 Stadiometer (Seca, United Kingdom). Weight was measured with light clothing using a Seca 813 digital electronic weighing scale (Seca, United Kingdom). Both WC and HC were measured with a non-elastic Seca measuring tape (Seca 201, Seca, UK), to the nearest millimeter. A portable body composition analyzer (Tanita SC-240 MA, Body Composition Analyser, Tanita Europe B.V., The Netherlands) was used to measure % BF. BMI was calculated as baseline body weight divided by height squared (kilograms per square meter). WHR was calculated as WC divided by HC (WC/HC). BMI was classified according to the World Health Organization criteria (26). For this study, adiposity parameters examined were BMI, WHR as measure of central adiposity, and % BF.

### SNP Selection and Genotyping

Genomic DNA from blood samples was extracted using GeneAll DNA Extraction Kits (Qiagen, Hilden, Germany). The quality of the extracted DNA was such that the absorbance ratios of at least 1.8 was attained for both 260/280 and 260/230 readings. All DNA samples and duplicates were diluted to 10 and 20 ng/µL, respectively, before being transferred to the respective wells. Quality controls included a blank and five duplicates.

Non-synonymous single-nucleotide polymorphisms that were reported to alter the amino acid sequence of protein candidate genes with multiple allele frequency (MAF) of more than 0.05 (except PPARG rs1800571) and have been previously associated with obesity were selected from the NCBI SNP database. A total of 11 nsSNPs within 10 candidate genes, namely, ADRB2 (rs1042714), ADRB3 (rs4994), FABP2 (rs1799883), GHRL (rs696217 and rs4684677), IGFBP1 (rs4619), LEPR (rs1805094), MC3R (rs3827103), PPARG (rs1800571), SHBG (rs6259), and VDR (rs2228570) were genotyped (see Table S1 in Supplementary Material for MAF and call rate). Genotyping was performed using a Sequenom MassARRAY platform with iPLEX GOLD chemistry (Sequenom, San Diego, CA, USA) following the manufacturer’s protocols. The MassARRAY system was created based on the technology of matrix-assisted laser desorption/ionization time-of-flight mass spectrometry.

### Statistical Analysis

Power calculation was performed using QUANTO (version 1.2.4, University of Southern California, Los Angeles, CA, USA). An estimated sample size of 1,059 would provide 80% power at an alpha of 0.05. The sample from MyHeARTs were sufficient to carry out further statistical analysis as pooled participants. For interaction analysis, we included all participants (case-only approach) and an estimated sample of 1,116 could achieve 80% power to detect gene–environment interaction in a model whereby the relative efficiency = 1 and *p* = 0.05 (27).

Continuous variables were presented as means with SDs, while categorical variables were presented as frequency and percentages. Variables were tested for normal distribution using the Kolmogorov–Smirnov test and further assessed using the *Q*–*Q* plots. The spread of data was obtained from the data itself. BMI and % BF did not conform to normal distribution and hence log transformation was performed for regression analysis. Comparisons of categorical variables were performed using χ^2^ test while the Students’ *T*-test and Mann–Whitney *U* test were used to compare continuous variable between those who were physically active and those inactive.

For each SNP, missing values were not imputed for all analyses. Genotype distribution was assessed for Hardy–Weinberg equilibrium (HWE) by using the χ^2^ test. A *p* value of more than 0.05 signifies agreement with HWE. 4 nsSNPs (ADRB2 rs1042714, IGFBP1 rs4619, GHRL rs4684677, and PPARG rs1800571) deviated from the HWE and were excluded from the final analyses (Table S1 in Supplementary Material). SNPs with very low frequencies of homozygote variants (*n* < 10) were also excluded from further analysis: LEPR rs1805094 and SHBG rs6259 (Table S1 in Supplementary Material). Unweighted genetic risk score (GRS) were calculated using the method described by Reddon et al. whereby the risk alleles of the five chosen nsSNPs were summed (each risk allele was scored as 1 and the other allele as 0) (19). The GRS scores could range from 0 to 10.

Linear regression using the generalized linear model (GLM) was used to examine the associations between PA and adiposity parameters (log transformed BMI, WHR, and log transformed % BF) using univariate and multivariate analysis adjusting for gender, ethnicity, and pubertal status. The GLM was also used to examine the association between genotypes of selected nsSNPs (homozygous, heterozygous, and homozygous variant) and adiposity parameters adjusting for covariates above. For WHR, the model included BMI as additional covariates. GLM was used to examine the interactions between individual nsSNPs, measured as additive allele (0 = no risk allele, 1 = 1 risk allele, and 2 = 2 risk alleles) and PA (total PAQ-C score) on logBMI, WHR, and log % BF. Interactions were examined (1) as unadjusted model (nsSNP × PA) and (2) by including a product term (PAQ-C scores × nsSNPs) adjusted for gender, ethnicity, pubertal stages, PAQ-C scores, and nsSNP.

The association between combined GRS and adiposity parameters and its interaction with PA were also examined using the same GLM model as described above. Associations and interaction effect were presented as beta (β) with 95% confidence interval (CI). To correct for testing for multiple adiposity parameters and SNPs, the false discovery rate (FDR) was calculated using Benjamini–Hochberg procedure (28). The FDR chosen was 0.25 and a corrected *p-*value of less than 0.025 was considered as statistically significant. Statistical analysis was performed using SPSS 24.0 software (IBM SPSS Statistics, NY, USA).

## Results

### Characteristics of the MyHeARTs Participants

Of the 1,151 15-year-old adolescents, 61% were females, 79% were of Malay ethnicity, and 62% had reached puberty (Table 1). Based on their PAQ-C scores, 48% were considered physically active while more than half were inactive. It was found that 863 adolescents (76%) were thin or of normal weight while 277 (24%) were classified as overweight or obese with no significant differences found across PA levels. Those who were physically active have significantly lower WHR (*p* = 0.01) and % BF (*p* < 0.0001) compared to those who were inactive.

**Table 1:** Demographics and adiposity parameters of Malaysian adolescents from the Malaysian Health and Adolescents Longitudinal Research Team study comparing physically active and inactive participants.

Demographics and adiposity parameters	All participants	Physically active	Physically inactive	p*
	*n*(%) or median ± IQR	*n*(%) or median ± IQR	*n*(%) or median ± IQR	
*N*	1,151	566 (49%)	585 (51%)	
**Gender**				0.003
– Male – Female	438 (39%) 713 (61%)	294 (67%) 272 (38%)	146 (33%) 439 (62%)	
**Ethnicity (*n* = 1,150)**				0.31
– Malays – Chinese – Indians – Others	905 (79%) 84 (7%) 102 (9%) 59 (5%)	468 (49%) 46 (55%) 47 (48%) 24 (41%)	441 (51%) 38 (45%) 52 (53%) 35 (59%)	
**Pubertal status (*n* = 1,064)**				0.003
– Prepubertal/early pubertal – Pubertal	333 (28%) 731 (62%)	139 (42%) 347 (52%)	191 (58%) 377 (48%)	
**Body mass indices (BMI) classification (*n* = 1,140)**				0.78
– Thin/normal – Overweight/obese	863 (76%) 277 (24%)	421 (49%) 138 (50)	442 (51%) 139 (50%)	
BMI (kg/m^2^)	19.8 (5.8)	19.9 (5.6)	19.6 (5.7)	0.13
Waist–hip ratio (mean)	0.78 (0.06)	0.79 (0.06)	0.78 (0.06)	0.01
BF (%)	24.0 (15.8)	22.5 (19.6)	25.0 (12.7)	<0.0001

*Comparing physically active and inactive using χ^2^ test, Student’s T-test, and Mann–Whitney U test as appropriate.

### Association Between PA (PAQ-C Scores) and Adiposity Parameters

In unadjusted regression model, those with higher total PAQ-C score were associated with significantly increased WHR and reduced % BF compared to those who with lower scores (Table 2). After adjustment, only % BF remained significantly associated with PAQ-C scores. Higher PAQ-C scores were associated with reduced % BF (β = −0.44 [95% CI −0.72, −0.16] *p* = 0.002).

**Table 2:** Association of physical activity (Physical Activity Questionnaire for Older Children scores) and adiposity parameters presented as β with 95% confidence interval (CI) and *p*-value.

Adiposity parameters	Unadjusted		Adjusted	
	β (95% CI)	*p*	β (95% CI)	*p*
Body mass indices (BMI)^a^	–0.00 (–0.01, 0.01)	0.47	0.00 (–0.01, 0.01)	0.94
Waist–hip ratio^b^	0.01 (0.00, 0.01)	0.004	–0.00 (–0.01, 0.01)	0.23
% BF^a^	–3.79 (–4.86, –2.72)	<0.0001	–0.44 (–0.72, –0.16)	0.002

*^a^Adjusted for gender, ethnicities, and pubertal stage*.

*^b^Adjusted for gender, ethnicities, pubertal stage, and BMI*.

### Association Between nsSNPs/GRS and Adiposity Parameters

The genotype distribution of selected nsSNPs and its association with adiposity parameters in the MyHeARTs cohort are presented in Table 3. In the unadjusted models, homozygote variants of ADRB3 rs4994 and MC3R rs3827103 were associated with reduced % BF, while FABP2 rs1799883 and GHRL rs696217 were associated with increased % BF. In the multivariate analysis and after considering correction for multiple testing, only the CC genotype of ADRB3 rs4994 (β = –0.16 [95% CI –0.28, –0.05], corrected *p* = 0.01) and AA genotype of MC3R rs3827103 (β = –0.06 [95% CI –0.12, –0.00], *p* = 0.02) were significantly associated with % BF compared to TT and GG genotypes, respectively. No other significant associations were found between nsSNPs and adiposity parameters in this population.

**Table 3:** Association between selected non-synonymous single-nucleotide polymorphisms (nsSNPs) and adiposity parameters in Malaysian Health and Adolescents Longitudinal Research Team study adolescents presented as β with 95% confidence interval (CI) and *p* values [uncorrected and corrected (*q*)].

nsSNP	Genotype		Unadjusted						Adjusted					
		LgBMI			Waist–hip ratio (WHR)		% BF		LgBMI^a^		WHR^b^		% BF^a^	
							
		*n*	β (95% CI)	*p*	β (95% CI)	*p*	β (95% CI)	*p*	β (95% CI)	*p*(*q*)*	β (95% CI)	*p*(*q*)*	β (95% CI)	*p*(*q*)*
ADRB3 rs4994	TT	900	Reference		Reference		Reference		Reference		Reference		Reference	
	TC	216	–0.00 (–0.02, 0.01)	0.88	–0.00 (–0.01, 0.01)	0.62	–0.02 (–0.06, 0.03)	0.48	–0.00 (–0.02, 0.01)	0.71 (0.17)	–0.01 (–0.02, 0.00)	0.17 (0.08)	–0.01 (–0.05, 0.02)	0.49 (0.14)
	CC	18	–0.04 (–0.09, 0.00)	0.06	–0.00 (–0.03, 0.03)	1.00	–0.21 (–0.34, 0.07)	0.003	–0.04 (–0.09, 0.00)	0.08 (0.02)	–0.02 (–0.05, 0.01)	0.17 (0.08)	–0.16 (–0.28, –0.05)	<0.0001 (0.01)

FABP2 rs1799883	GG	778	Reference		Reference		Reference		Reference		Reference		Reference	
	GA	319	0.00 (–0.01, 0.02)	0.51	–0.00 (–0.01, 0.01)	0.88	0.02 (–0.02, 0.06)	0.25	0.00 (-0.01, 0.02)	0.77 (0.21)	0.00 (–0.01, 0.01)	0.74 (0.20)	–0.00 (–0.03, 0.03)	0.88 (0.24)
	AA	37	0.01 (–0.0, 0.04)	0.42	–0.01 (–0.03, 0.01)	0.51	0.14 (0.05, 0.23)	0.004	0.01 (–0.02, 0.04)	0.58 (0.17)	0.00 (–0.02, 0.03)	0.72 (0.18)	0.03 (–0.05, 0.11)	0.45 (0.13)

GHRL rs696217	GG	782	Reference		Reference		Reference		Reference		Reference		Reference	
	GT	311	0.00 (–0.01, 0.01)	0.81	–0.01 (–0.02, 0.00)	0.14	–0.00 (–0.04, 0.04)	0.89	–0.00 (–0.01, 0.01)	0.91 (0.25)	–0.00 (–0.01, 0.00)	0.36 (0.11)	–0.02 (–0.05, 0.01)	0.14 (0.05)
	TT	40	0.04 (0.01, 0.07)	0.01	0.01 (–0.01, 0.03)	0.21	0.11 (0.02, 0.20)	0.01	0.03 (–0.01, 0.06)	0.10 (0.05)	0.02 (–0.00, 0.04)	0.08 (0.02)	0.05 (–0.03, 0.12)	0.24 (0.09)

MC3R rs3827103	GG	643	Reference		Reference		Reference		Reference		Reference		Reference	
	GA	429	–0.00 (–0.02, 0.01)	0.49	0.00 (–0.01, 0.01)	0.97	–0.04 (–0.07, 0.01)	0.04	–0.00 (–0.02, 0.01)	0.50 (0.14)	–0.00 (–0.01, 0.01)	0.43 (0.12)	–0.02 (–0.05, 0.01)	0.16 (0.07)
	AA	68	–0.02 (–0.05, 0.00)	0.05	–0.01 (–0.02, 0.01)	0.30	–0.08 (–0.15, 0.01)	0.03	–0.02 (–0.04, 0.01)	0.14 (0.05)	–0.01 (–0.03, 0.01)	0.24 (0.09)	–0.06 (–0.12,–0.00)	0.05 (0.02)

Vitamin D receptor rs2228570	CC	407	Reference		Reference		Reference		Reference		Reference		Reference	
	TC	527	0.00 (–0.01, 0.01)	0.77	0.00 (–0.01, 0.01)	0.93	0.01 (–0.03, 0.05)	0.57	–0.00 (–0.01, 0.01)	0.80 (0.22)	0.00 (–0.01, 0.01)	0.52 (0.16)	0.01 (–0.03, 0.04)	0.73 (0.20)
	TT	207	0.00 (–0.02, 0.02)	0.97	–0.00 (–0.01, 0.01)	0.52	0.00 (–0.04, 0.05)	0.88	–0.00 (–0.02, 0.02)	0.83 (0.23)	–0.00 (–0.01, 0.01)	0.72 (0.18)	–0.00 (–0.04, 0.04)	0.85 (0.23)

Genetic risk score			–0.00 (–0.00, 0.00)	0.99	–0.00 (–0.00, 0.00)	0.25	0.00 (–0.01, 0.01)	0.81	–0.00 (–0.01, 0.00)	0.50 (0.14)	–0.00 (–0.00, 0.00)	0.29 (0.11)	–0.01 (–0.02, 0.00)	0.09 (0.04)

*^a^Model adjusted for gender, ethnicity, and pubertal stages*.

*^b^Model adjusted for gender, ethnicity, pubertal stages, and body mass indices (BMI)*.

*Corrected p value based on Benjamini–Hochberg procedure.

### Interaction Between nsSNPs and PA on Adiposity Parameters

In the unadjusted model, there were significant interactions found between ADRB3 rs4994, MC3R rs3827103, VDR rs2228570, and GRS and total PAQ-C scores on % BF (Table 4). However, in the adjusted model that included the main effects, significant interaction with PA on % BF were observed only with ADRB3 rs4994 (β = –0.05[–0.10,–0.01], *p* = 0.02) and combined GRS (β = –0.03[–0.04,–0.01], *p* = 0.01) with small effect size observed per unit increase.

**Table 4:** Interaction between selected non-synonymous single-nucleotide polymorphisms (nsSNPs) (additive allele model) and their combined genetic risk score (GRS) and physical activity (Physical Activity Questionnaire for Older Children scores) in Malaysian Health and Adolescents Longitudinal Research Team study adolescents presented as β and 95% confidence interval (CI) and *p* values (uncorrected and corrected *q*).

nsSNP	Unadjusted						Adjusted					
	LgBMI		Waist–hip ratio (WHR)		% BF		LgBMI^a^		WHR^b^		% BF^a^	
					
	β (95% CI)	*p*	β (95% CI)	*p*	β (95% CI)	*p*	β (95% CI)	*p*(q)*	β (95% CI)	*p*(*q*)*	β (95% CI)	*p*(*q*)*
ADRB3 rs4994	–0.00 (–0.01, 0.00)	0.17	–0.00 (–0.01, 0.00)	0.32	–0.04 (–0.04, –0.02)	<0.0001	–0.01 (–0.03, 0.00)	0.12 (0.11)	–0.00 (–0.01, 0.01)	0.76 (0.19)	–0.05 (–0.10, –0.01)	0.02 (0.02)
FABP2 rs1799883	0.00 (–0.00, 0.01)	0.57	–0.00 (–0.00, 0.00)	0.26	–0.01 (–0.02, 0.00)	0.04	0.01 (–0.01, 0.02)	0.50 (0.17)	–0.00 (–0.01, 0.01)	0.90 (0.20)	–0.00 (–0.04, 0.04)	1.00 (0.21)
GHRL rs696217	0.00 (–0.00, 0.01)	0.37	0.00 (–0.00, 0.01)	0.29	–0.02 (–0.03, –0.01)	0.03	–0.01 (–0.02, 0.01)	0.40 (0.15)	–0.01 (–0.01, 0.01)	0.50 (0.17)	–0.02 (–0.06, 0.02)	0.28 (0.14)
MC3R rs3827103	–0.00 (–0.01, 0.01)	0.13	–0.00 (–0.00, 0.00)	0.57	–0.03 (–0.04, –0.02)	<0.0001	–0.01 (–0.02, 0.01)	0.20 (0.13)	–0.01 (–0.02, 0.00)	0.05 (0.07)	–0.04 (–0.07, –0.00)	0.04 (0.05)
Vitamin D receptor rs2228570	–0.00 (–0.00, 0.00)	0.71	0.00 (–0.00, 0.01)	0.41	–0.02 (–0.03, –0.01)	<0.0001	–0.01 (–0.02, 0.00)	0.13 (0.12)	–0.01 (–0.02, 0.00)	0.11 (0.10)	–0.04 (–0.07, –0.00)	0.03 (0.04)
GRS	–0.00 (–0.00, 0.00)	0.57	–0.00 (–0.00, 0.00)	0.68	–0.01 (–0.02, –0.01)	<0.0001	–0.01 (–0.01, 0.00)	0.05 (0.07)	–0.01 (–0.01, 0.00)	0.04 (0.05)	–0.03 (–0.04,–0.01)	<0.0001 (0.01)

*^a^Model include interaction term (SNP × PA), SNP, PA, gender, ethnicity, and pubertal stage*.

*^b^Model include interaction term (SNP × PA), SNP, PA, gender, ethnicity, pubertal stage, and body mass indices (BMI)*.

*Corrected p value based on Benjamini–Hochberg procedure References.

## Discussion

Of the MyHeARTs participants, almost 25% were considered overweight and obese and 48% were physically inactive. Higher PA scores were significantly associated with reduced % BF in our adolescents. % BF was shown to be more influenced by nsSNPs rather than measurement of BMI or circumferences with CC genotype of ADRB3 rs4994 and AA genotype of MC3R rs3827103 shown to be associated with small but significant protective effect on this adiposity measure. Significant interactions with PA on adiposity measures were demonstrated where ADRB3 rs4994 and combined GRS shown to interact with PA on % BF with overall protective effect.

Previous study on Malaysian adolescents from the first wave of MyHeARTs cohort demonstrated inverse correlation between PA scores and % BF, as well as waist circumference and BMI (4). In addition, it was shown that the levels of PA in adolescents who were obese were low, whereby less than a third were considered as physically active. The protective effect of PA on adiposity parameters represents an opportunity for early intervention in adolescents, in view of the high prevalence of obesity in this country (1). Of concern, significant downward trends of PA were shown in Malaysian girls in the 2-year follow-up of the same cohort accompanied by concurrent % BF increment (29). Thus, effective policies to promote PA in this age group are warranted with interventions to be planned at all levels of the community (30).

The protective effects of the MC3R rs3827103 on body fat was particularly evident in subjects with AA genotype. MC3R may have a role in inhibiting energy storage and lowering food efficiency—the body’s ability to convert food to adiposity (31). By contrast, a study among obese children in Thailand on this nsSNPs showed no significant association with adiposity (32). We observed that the ADRB3 rs4994 were associated with changes in % BF where CC homozygotes had protective effects. ADRB3 rs4994 has been reported to be associated with body fat distribution in Koreans (33). The ADRB3 rs4994 (Trp64Arg) polymorphism may exert functional effect on the β3-adrenergic receptor which are involved in regulating lipolysis in adipose tissues and have a role in energy expenditure (34). Kurokawa et al. in his meta-analysis demonstrated that the ADRB3 rs4994 were associated with obesity in East Asian rather than in Caucasians (14). However, studies on this SNPs yielded inconsistent results (35) and thus it has been suggested that this SNP may demonstrate ethnic heterogeneity (36).

We did not observe significant association between GHRL rs696217 polymorphisms and any adiposity markers. The GHRL rs696217 or leu72met was one of the most commonly studied SNPs of the preproghrelin gene and lies outside the region where mature ghrelin product is encoded (37), hence its functional significance remains uncertain. Nevertheless, as ghrelin is an orexigenic peptide, this mutation might produce an increased expression or gain in function of the peptide. This is thought to eventually influence ghrelin secretion and/or activity which is important in regulating satiety, food intake and energy balance in human (38). Studies in both children and adult populations demonstrated associations between this nsSNP and adiposity parameters such as BMI (39–41), visceral fat areas (42) and HC (43) while others could not demonstrate significant associations (44, 45). Furthermore, we did not observe any significant association between FABP2 rs1799883 and VDR rs2228570 polymorphisms and any adiposity markers. Their association with obesity-related parameters have been previously studied in different populations (14, 31–33, 46–50) with differing results. This could be possibly due to the genetic heterogeneity or differences between populations in terms of environmental factors that influence phenotypic expression of the gene variants.

ADRB3 rs4994 and combined GRS of these nsSNPs was shown to interact with PA on % BF while no significant interactions were found for other adiposity parameters. A study by Marti et al. suggested that ADRB3 rs4994 interacts with PA whereby it is a risk factor for obesity among inactive but not in physically active individuals (51). In contrast to our study, the Finnish Diabetes Prevention Study found that GHRL rs696217 modified the effect of moderate-to-vigorous PA on weight and WC in their overweight cohort (18). Studying the effect of gene and PA interaction using adiposity parameters such as % BF instead of the commonly used measures of BMI may potentially uncover novel susceptible SNPs compared to when using BMI in children and adolescent. In the Asia Pacific region, the SCOPE study on 6-year-old children New Zealander of Caucasian origin found that SNPs such as OLFM4-9568856, GNPDA2-rs10938397, CLOCK-rs4864548, and LEPR-1045895 showed genotype differences with PA when comparing % BF (20). This New Zealand study suggested that children who maybe genetically predisposed to obesity could benefit from higher levels of PA to improve their body fat profile. Although our study differs from the New Zealand in terms of ethnicities of children involved, the key message remains true. Targeting at-risk children and adolescents at time when their PA levels are still modifiable by interventions represents opportunity to reduce obesity in the general population. Yet, it is worth noting that meta-analysis of randomized controlled trial could not find the differential effects of the FTO SNPs, the most commonly studied SNPs and environment interaction, on weight loss interventions (52). This raises the question on the clinical significance of gene and environment interaction on interventions targeting obesity. Utilization of multiple gene models is necessary in further studies to consider exercise intervention as a preventive health strategy which is clinically relevant to predict the individual response.

To our knowledge, this is the first adolescent study on adiposity in Malaysia that has included both genetic and environment components using comprehensive anthropometric assessments. The MyHeARTs recruited adolescents from different states in Malaysia encompassing both urban and rural settings and different socioeconomic groups. This longitudinal cohort provides a wealth of information on adolescents’ health and lifestyle in this region of the world. As this study is part of a longitudinal cohort, there were limitations in terms of participants included. This cohort has low representative of the Chinese ethnic groups and therefore analyses were only performed in pooled participants. Thus, the sample is rather heterogeneous in terms of ethnicity. We included ethnicity as covariates in the association study, however, replication of this study using higher number of adolescents from these ethnicities of similar ancestries should be planned to allow stratification of genetic risk according to ethnicity. Besides twins and siblings, family relationship between participants were not able to be ascertained. Physical activities were self-reported by students rather than measured by devices and thus may still be subjected to recall bias. However, the PAQ-C is a validated tool that has good internal consistency and acceptable validity in Malaysia (4). We did not include dietary or nutritional information to account for the effect of these environmental factors in our models.

Selection of only nsSNPs to be studied has its own limitations. NsSNPs generally have lower frequency than synonymous SNP; hence, there lies the probability of false positive in a small sample of general population. Six out of 11 nsSNPs genotyped were not included in the final analysis due to deviation from HWE or low frequency of homozygous variants which may reflect differences in our population compared to others. The number of adolescents in the extreme phenotype (morbid obesity) was small and therefore was not studied separately. This group may represent true risk of genetic and environment interactions especially when considering non-synonymous mutation and hence future study using methods such as exome sequencing should be considered.

In conclusion, higher PA score was associated with reduced % BF in our Malaysian adolescents. Only ADRB3 rs4994 and MC3R rs3827103 were shown to be associated with reduced % BF, while the other nsSNPs were not found to be associated with adiposity measures in our population. Significant interactions with PA were found for ADRB3 rs4994 and combined GRS on % BF rather than measurements of weight or circumferences. These findings represent prospects for molecular studies and opportunity to incorporate genetic and lifestyle factors in elucidating the contributions to obesity problem and planning of appropriate intervention for Malaysian adolescents, especially targeting at the body fat. Further follow-up from this longitudinal cohort should examine changes in the interaction of PA and SNPs on % BF over time.

## Ethics Statement

The genetic part of the study was carried out in accordance with the recommendations of Guidelines on the Ethical Practice in Human Genetics and Human Genomic Research, Universiti Sains Malaysia (USM) Human Research Ethics Committee with written informed consent from all subjects (53). All subjects gave written informed consent in accordance with the Declaration of Helsinki. The protocol was approved by the ethics committees of the University of Malaya Medical Centre (MEC Ref. No: 896.34).

## Author Contributions

All authors are involved in the conception or design of the work. HM, NM, NZ, TS, MJ, ZM, and MM are involved in acquisition, analysis, and interpretation of data for the work; NZ and NM drafted the manuscript, and all authors were involved in revising the manuscript and final approval of the submitted manuscript.

## Conflict of Interest Statement

The authors declare that the research was conducted in the absence of any commercial or financial relationships that could be construed as a potential conflict of interest.
