# Supplementary material for: Non-Synonymous Single-Nucleotide Polymorphisms and Physical Activity Interactions on Adiposity Parameters in Malaysian Adolescents
Source: Front Endocrinol (Lausanne). 2018 Apr 27;9:209. doi: 10.3389/fendo.2018.00209 (PMC5934415; doi:10.3389/fendo.2018.00209)
Supplement: Supplementary file 1 [file table_1.docx]

Supplementary Table 1: Characteristics of nsSNPs selected for genotyping

| **NO** | **Gene** | **SNP ID** | **Variation** | **Genotype** | **Frequency** | **HWE** | **MAF** | **CALL-RATE** |
| --- | --- | --- | --- | --- | --- | --- | --- | --- |
| 1 | ADRB2 | rs1042714 | Glu27Gln | CC | 985 | <0.00001 | 0.48 | 91.8 |
|  |  |  |  | GC | 52 |  |  |  |
|  |  |  |  | GG | 19 |  |  |  |
| 2 | ADRB3 | rs4994 | Trp64Arg | TT | 900 | 0.23 | 0.01 | 98.6 |
|  |  |  |  | TC | 216 |  |  |  |
|  |  |  |  | CC | 18 |  |  |  |
| 3 | FABP2 | rs1799883 | Thr55Ala | GG | 778 | 0.54 | 0.25 | 98.6 |
|  |  |  |  | GA | 319 |  |  |  |
|  |  |  |  | AA | 37 |  |  |  |
| 4 | GHRL | rs696217 | Leu72Met | GG | 782 | 0.19 | 0.08 | 98.5 |
|  |  |  | Leu71Met | GT | 311 |  |  |  |
|  |  |  |  | TT | 40 |  |  |  |
| 5 | GHRL | rs4684677 | Gln89Leu | TT | 1099 | 0.02 | 0.06 | 99 |
|  |  |  |  | TA | 40 |  |  |  |
|  |  |  |  | AA | 0 |  |  |  |
| 6 | IGFBP1 | rs4619 | Ile253Met | AA | 322 | 0.002 | 0.38 | 96.1 |
|  |  |  |  | GA | 500 |  |  |  |
|  |  |  |  | GG | 284 |  |  |  |
| 7 | LEPR | rs1805094 | Lys656Asn | GG | 1050 | 0.56 | 0.14 | 97.9 |
|  |  |  |  | CG | 74 |  |  |  |
|  |  |  |  | CC | 2 |  |  |  |
| 8 | MC3R | Rs3827103 | Val44lle | GG | 643 | 0.75 | 0.25 | 99.1 |
|  |  |  |  | GA | 429 |  |  |  |
|  |  |  |  | AA | 68 |  |  |  |
| 9 | PPARG | rs1800571 | Pro85Gln | CC | 1137 | NA | NA | 98.8 |
| 10 | SHBG | rs6259 | Asp298Asn | GG | 1075 | 0.31 | 0.07 | 99.2 |
|  |  |  | Asp183Asn | GA | 64 |  |  |  |
|  |  |  |  | AA | 2 |  |  |  |
| 11 | VDR | rs2228570 | Met1Thr | CC | 407 | 0.11 | 0.33 | 99.2 |
|  |  |  |  | TC | 527 |  |  |  |
|  |  |  |  | TT | 207 |  |  |  |

HWE:Hardy-Weinburg equilibrium

MAF: Minor allele frequency
